# Tolerability of Lithium: A Naturalistic Discontinuation Study in Older Adults (≥60 Years)

**DOI:** 10.1093/geroni/igab046.349

**Published:** 2021-12-17

**Authors:** Marinke Flapper, Els van Melick, Jos van Campen, Natasja Schutter, Rob Kok

**Affiliations:** 1 Onze Lieve Vrouwe Gasthuis Amsterdam, Amsterdam, Drenthe, Netherlands; 2 Hospital Reinier de Graeff, Delft, Zuid-Holland, Netherlands; 3 Hospital OLVG, Hospital OLVG, Noord-Holland, Netherlands; 4 Arkin Ouderen, Amsterdam, Noord-Holland, Netherlands; 5 Parnassia, Den Haag, Zuid-Holland, Netherlands

## Abstract

Lithium is one of the most effective treatment options in both bipolar disorder and treatment-resistant depression. The use of lithium in older adults declined during the last decades, probably resulting in undertreatment of older adults. To investigate how well lithium is tolerated in old age, we aimed to determine the frequency, reasons and possible predictors of discontinuation due to adverse effects in a cohort of hospitalized adults aged 60 years or older who had started with lithium.

We performed a retrospective cohort study based on chart reviews. Participants were in treatment at Parnassia Group at The Hague, the Netherlands. After inclusion (between January 2010 and December 2016), participants were followed until April 2017, when we performed data extraction and analysis.

In our sample of 135 patients (median age 69 years, median follow-up duration 18 months), 49 (36.3%) participants discontinued lithium. Only a minority (11 (8.1%)) of the participants discontinued solely due to adverse effects. The majority discontinued lithium due to psychiatric (18,5%) reasons, (most commonly mentioned within this subgroup: lack of effectiveness and non-compliance) or a combination of reasons (7.4%). None of the factors we studied (age, gender, Charlson Comorbidity Index (CCI), polypharmacy, renal function and neurological history) were significantly associated with discontinuation due to adverse effects.

The frequency of lithium discontinuation in our cohort was in range with frequencies reported in younger patients. Older age itself should not be a reason to withhold lithium treatment.

